# Adjustment of Dysregulated Ceramide Metabolism in a Murine Model of Sepsis-Induced Cardiac Dysfunction

**DOI:** 10.3390/ijms18040839

**Published:** 2017-04-15

**Authors:** Ha-Yeun Chung, Anna S. Kollmey, Andrea Schrepper, Matthias Kohl, Markus F. Bläss, Sebastian N. Stehr, Amelie Lupp, Markus H. Gräler, Ralf A. Claus

**Affiliations:** 1 Center for Sepsis Control and Care, Jena University Hospital, Am Klinikum 1, 07747 Jena, Germany; Ha-yeun.Chung@med.uni-jena.de (H.-Y.C.); sophia.kollmey@web.de (A.S.K.); Andrea.Schrepper@med.uni-jena.de (A.S.); sebastian.stehr@medizin.uni-leipzig.de (S.N.S.); markus.graeler@med.uni-jena.de (M.H.G.); 2 Department of Anesthesiology and Intensive Care, Jena University Hospital, Am Klinikum 1, 07747 Jena, Germany; blae@hs-furtwangen.de; 3 Department of Cardiothoracic Surgery, Jena University Hospital, Am Klinikum 1, 07747 Jena, Germany; 4 Institute of Precision Medicine, Furtwangen University, 78054 Villingen-Schwenningen, Germany; matthias.kohl@stamats.de; 5 Department of Anesthesiology and Intensive Care, University of Leipzig, Liebigstrasse 20, 04103 Leipzig, Germany; 6 Institute of Pharmacology and Toxicology, Jena University Hospital, Drackendorfer Straße 1, 07747 Jena, Germany; amelie.lupp@med.uni-jena.de

**Keywords:** acid sphingomyelinase, de novo synthesis, ceramide, desipramine, sepsis, cardiac dysfunction

## Abstract

Cardiac dysfunction, in particular of the left ventricle, is a common and early event in sepsis, and is strongly associated with an increase in patients’ mortality. Acid sphingomyelinase (SMPD1)—the principal regulator for rapid and transient generation of the lipid mediator ceramide—is involved in both the regulation of host response in sepsis as well as in the pathogenesis of chronic heart failure. This study determined the degree and the potential role to which SMPD1 and its modulation affect sepsis-induced cardiomyopathy using both genetically deficient and pharmacologically-treated animals in a polymicrobial sepsis model. As surrogate parameters of sepsis-induced cardiomyopathy, cardiac function, markers of oxidative stress as well as troponin I levels were found to be improved in desipramine-treated animals, desipramine being an inhibitor of ceramide formation. Additionally, ceramide formation in cardiac tissue was dysregulated in SMPD1^+/+^ as well as SMPD1^−/−^ animals, whereas desipramine pretreatment resulted in stable, but increased ceramide content during host response. This was a result of elevated de novo synthesis. Strikingly, desipramine treatment led to significantly improved levels of surrogate markers. Furthermore, similar results in desipramine-pretreated SMPD1^−/−^ littermates suggest an SMPD1-independent pathway. Finally, a pattern of differentially expressed transcripts important for regulation of apoptosis as well as antioxidative and cytokine response supports the concept that desipramine modulates ceramide formation, resulting in beneficial myocardial effects. We describe a novel, protective role of desipramine during sepsis-induced cardiac dysfunction that controls ceramide content. In addition, it may be possible to modulate cardiac function during host response by pre-conditioning with the Food and Drug Administration (FDA)-approved drug desipramine.

## 1. Introduction

Sepsis-induced cardiac dysfunction (SCD) is a common phenomenon in sepsis/septic shock, leading to an increase in mortality [1,2]. Patients typically present with diastolic dysfunction and decreased ejection fraction (EF) in transesophageal or transthoracic echocardiography (TTE) [3]. Molecular mechanisms and associated pathophysiological alterations in the myocardium during host response still largely remain unclear. Observational studies have demonstrated an association between increased troponin I levels, cardiac dysfunction and unfavourable outcome in sepsis [4,5,6]. Sepsis is defined as life-threatening organ dysfunction due to a dysregulated host response to infection [7]. SCD is present in almost 50% of sepsis cases [8] and has been documented as one of the major predictors of morbidity and mortality related to sepsis. Although SCD can be reversible [9], its occurrence increases the mortality of septic patients to up to 70% [10,11].

The plasma-secreted isoform of acid sphingomyelinase (SMPD1) is a conserved enzyme in cellular stress response playing a detrimental role in sepsis and remote organ failure [12]. Enhancement of activity and increase in concentration in plasma correlates with disease severity of septic patients and discriminates with respect to outcome [13,14,15]. Patients with chronic heart failure also have increased activity, associated with decreased vascular functional capacity and poor long-term outcome [16]. SMPD1 is of major significance as an effector of rapid and transient ceramide generation with differing lengths of acylated fatty acids by hydrolysis of inert, membrane-embedded sphingomyelin [17,18]. Besides breakdown of sphingomyelin, ceramides can also be generated by isoforms (e.g., neutral sphingomyelinase), a salvage pathway recycling sphingosine/sphingosine-1-phosphate, as well as by de novo synthesis, resulting in complex metabolism and signalling [19]. However, there is evidence concerning the role of generated intermediate-chain ceramides in assembling lipid rafts to initiate the reorganization of receptor proteins in the outer leaflet of endothelial membranes [20]. Short-chained ceramides have been described as second messengers [21,22]. All of these specimens control cell proliferation, differentiation and apoptosis [23,24]. Ceramides regulate the activation of volume-sensitive chloride current in cardiomyocytes which control cardiac electro-mechanical activity and cell volume as well as apoptosis [25] and contribute to lipotoxic cardiomyopathy [26,27]. Hence, functional inhibitors of SMPD1 (FIASMA) such as amitriptyline protect cardiomyocytes from apoptosis and maintain functional activity in an ex vivo cardioplegia and reperfusion model [28].

Amitriptyline and desipramine and other FIASMA belong to a group of compounds which are trapped in lysosomes due to biophysical properties [29]. FIASMA initiate a proteolytic breakdown of numerous enzymes including SMPD1 [29], but it has been demonstrated that these compounds also inactivate other enzymes relevant for ceramide inactivation such as ceramidases [30,31]. Most of the entities named FIASMAs are included in the World Health Organization (WHO) drug list of approved and essential medicines such as Alzheimer’s disease, major depression, radiation- and chemotherapy-induced apoptosis and endotoxic shock syndrome. About half of them are listed in the US FDA-approved drug list, characterized not only by low toxicity but also by records of clinical experience for decades. FIASMAs have a number of favourable properties in the context of clinical application, suggesting the potential for rapid advancement into preclinical and/or clinical trials [29,31].

We investigated the role of SMPD1 and associated ceramide generation as a detrimental mediator of cardiomyocyte integrity and function. In addition, the effects of desipramine in modulating ceramide metabolism and its protective effect in a murine model of severe sepsis were examined. Polymicrobial sepsis was induced by peritoneal contamination and infection (PCI) [32], and laboratory as well as clinical parameters of sepsis-induced cardiac dysfunction correlating with ceramide generation were evaluated.

## 2. Results

Previous studies demonstrated increased SMPD1 activity in patients with sepsis as well as with chronic heart failure, both correlating to disease severity [13,15,16]. Therefore, we intended to clarify the role of SMPD1 in contributing to cardiac ceramide generation during sepsis using mice in which either the enzyme was totally absent through genetic knock-out (SMPD1^−/−^) or partially inhibited by a FIASMA (desipramine, d) (dSMPD1^+/+^ as well as dSMPD1^−/−^), compared to SMPD1^+/+^.

### 2.1. (Pre-)treatment with Desipramine Improves Cardiac Function in Polymicrobial Sepsis

**Figure 1:**
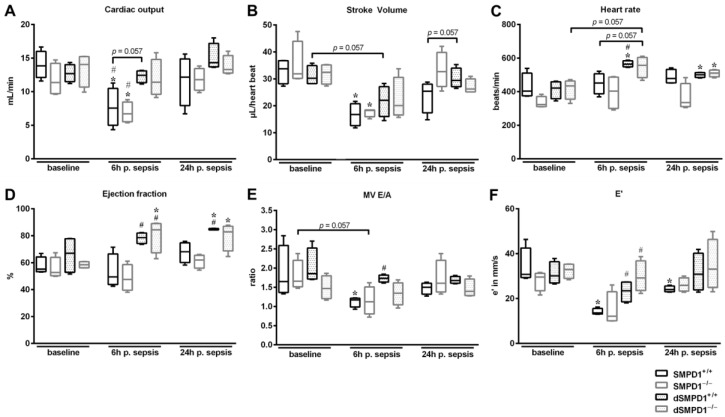
Evaluation of desipramine treatment on cardiac function. Transthoracic echocardiography measurements at 6 and 24 h following sepsis (*n* = 4 per strata/time point). (**A**) Cardiac output was calculated by stroke volume (**B**) and heart rate (**C**). SMPD1^+/+^ and SMPD1^−/−^ animals displayed impaired cardiac output, whereas measurements of desipramine-pretreated (d) strata demonstrated no changes during sepsis; (**D**) Ejection fraction (EF) was significantly increased in dSMPD1^+/+^ and dSMPD1^−/−^ animals compared to SMPD1^+/+^ at 6 h following polymicrobial sepsis; (**E**) Data of mitral valve E/A (MV E/A) as well as (**F**) E’ revealed impaired diastolic ventricular function in SMPD1^+/+^ animals and SMPD1^−/−^ animals (*p* = 0.057) following polymicrobial sepsis, but showed less pronounced altered function in dSMPD1^+/+^ as well as in dSMPD1^−/−^. * *p* ≤ 0.05 versus corresponding baseline values; # *p* ≤ 0.05 versus SMPD1^+/+^ at corresponding time points. Abbreviations: p. sepsis = post sepsis.

In both untreated strata (SMPD1^+/+^ and SMPD1^−/−^ animals) PCI resulted in SCD being significantly decreased (Figure 1A), whereas desipramine-pretreated strata (dSMPD1^+/+^ and dSMPD1^−/−^) revealed unchanged cardiac output levels. Similar results were measured in stroke volume (Figure 1B). Heart rate (Figure 1C) was significantly increased in dSMPD1^+/+^ animals as compared to untreated SMPD1^+/+^ littermates. No differences in ejection fraction (Figure 1D) in SMPD1^+/+^ and SMPD1^−/−^ were observed at 6 and 24 h following sepsis, whereas desipramine-pretreated (dSMPD1^+/+^) animals displayed a significantly improved EF at 6 and 24 h (baseline: 66.9 (IQR 52.8–77.8) vs. 6 h: 78.65 (IQR 74.2–82.1) vs. 24 h: 84.5 (IQR 84.3–85.4), *p* ≤ 0.05 vs. baseline as well as untreated SMPD1^+/+^) during sepsis. Pretreatment (dSMPD1^−/−^) resulted in increased EF at 6 h (baseline: 58.47% ± 2.51% vs. 80.24% ± 12.19%, *p* ≤ 0.05 vs. baseline as well as untreated SMPD1^+/+^) and at 24 h (79.52 ± 10.34%, *p* ≤ 0.05 vs. baseline).

In sepsis, diastolic dysfunction is correlated to enhanced mortality rate [2]. Therefore, E’ as well as MV E/A are common parameters to measure dysfunction in capability of ventricular dilatation. The parameter E’ (Figure 1F) revealed significant impaired diastolic function in SMPD1^+/+^ at 6 h (baseline: 34.23 ± 8.12 vs. 14.08 ± 1.43 mm/s, *p* ≤ 0.05) and 24 h (24.23 ± 1.34 mm/s, *p* ≤ 0.05) as compared to baseline values following sepsis, whereas diastolic dysfunction was less pronounced in both pretreated strata. Both SMPD1^+/+^ (baseline: 1.87 ± 0.68 vs. 1.12 ± 0.13, *p* ≤ 0.05) and SMPD1^−/−^ animals (baseline: 1.79 ± 0.4 vs. 1.15 ± 0.37, *p* = 0.057) displayed a reduction of MV E/A at 6 h following polymicrobial sepsis as compared to baseline values (Figure 1E). dSMPD1^+/+^, as well as dSMPD1^−/−^ displayed no changes during sepsis at both time points, respectively, and significantly higher values were shown at 6 h in dSMPD1^+/+^ (1.73 ± 0.1, *p* ≤ 0.05) as compared to SMPD1^+/+^ at a similar time point.

### 2.2. Desipramine Treatment Reduces Oxidative Stress in the Heart during the Acute Phase of Sepsis

**Figure 2:**
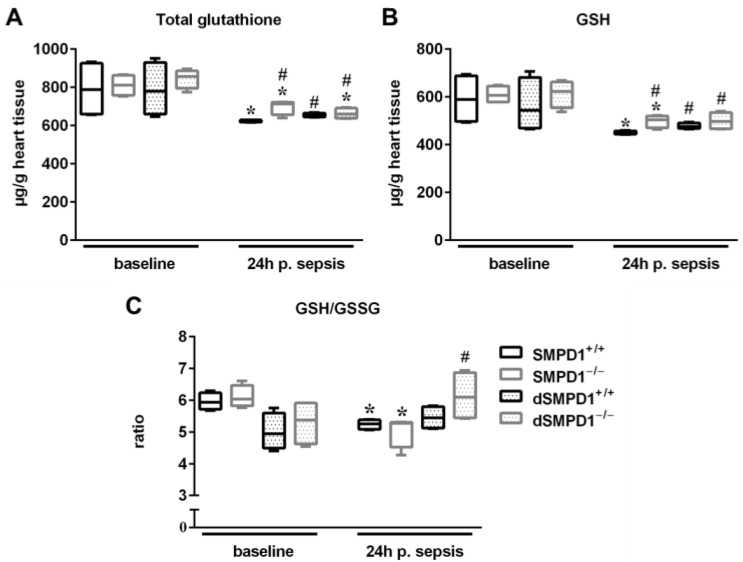
Reduced oxidative stress in desipramine-pretreated animals. Measurement of reduced glutathione (GSH) and glutathione disulfide (GSSG) as surrogates of oxidative stress in cardiac tissue homogenates (*n* = 4 animals per strata/time point). (**A**) Total glutathione and (**B**) GSH was decreased in all four strata, however changes were less pronounced in dSMPD1^+/+^, dSMPD1^−/−^ and SMPD1^−/−^ animals compared to SMPD1^+/+^ at 24 h following septic insult; (**C**) GSH/GSSG ratio decreased in SMPD1^+/+^ and SMPD1^−/−^ animals, but remained unchanged in dSMPD1^+/+^ and dSMPD1^−/−^ strata as compared to baseline values following sepsis. * *p* ≤ 0.05; versus corresponding baseline values; # *p* ≤ 0.05 versus SMPD1^+/+^ at corresponding time points. Abbreviations: p. sepsis = post sepsis.

Ceramides are key mediators of cellular function, including oxidative stress and initiation of apoptosis in cardiomyocytes [33], which prompted us to measure oxidative stress in heart homogenates of different strata. Glutathione levels decreased significantly in SMPD1^+/+^ from baseline to 24 h following sepsis (baseline: 787.2 (IQR 658.9–925.6 vs. 623.0 (IQR 616.7–628.5) µg/g, *p* ≤ 0.05; Figure 2A). This decrease was also observed in SMPD1^−/−^ (baseline: 811.4 (IQR 757.5–861.7) vs. 712.7 (IQR 655.8–721.7) µg/g, *p* ≤ 0.05) as well as dSMPD1^−/−^ animals (baseline: 855.5 (IQR 793.5–885.8) vs. 659.8 (IQR 637.2–690.0) µg/g, *p* ≤ 0.05), but not in SMPD1^+/+^ following desipramine pretreatment. Interestingly, all strata resulted in significantly elevated total glutathione levels in the acute phase as compared to SMPD1^+/+^.

Glutathione levels (Figure 2B) decreased significantly in untreated SMPD1^+/+^ at 24 h following sepsis as compared to corresponding baseline animals (baseline: 588.5 (IQR 496.3–687.8) vs. 451.1 (IQR 443.8–457.0) µg/g, *p* ≤ 0.05). In addition, SMPD1^−/−^ animals also exhibited a significant decrease (baseline: 605.9 (IQR 578.4–644.8) vs. 502.9 (IQR 470.1–519.5) µg/g heart), whereas significantly higher GSH levels were observed as compared to untreated SMPD1^+/+^. Strikingly, dSMPD1^+/+^ (475.5 (IQR 466.7–490.2) µg/g, *p* ≤ 0.05) and dSMPD1^−/−^ animals (496.3 (IQR 466.1–534.3) µg/g, *p* ≤ 0.05) also revealed significantly higher GSH levels compared to SMPD1^+/+^ in the acute phase.

The GSH/GSSG ratio (Figure 2C) decreased in the untreated SMPD1^+/+^ group at 24 h following sepsis (baseline: 5.9 (IQR 5.7–6.2) vs. 5.3 (IQR 5.1–5.4), *p* ≤ 0.05) as well as in the untreated SMPD1^−/−^ group (baseline: 6.0 (IQR 5.8–6.5) vs. 5.3 (IQR 4.5–5.3), *p* ≤ 0.05). However, desipramine pretreatment of SMPD1^+/+^ (baseline: 4.9 (IQR 4.5–5.6) vs. 5.4 (5.1–5.7), not significant (n.s.)) and SMPD1^−/−^ animals (baseline: 5.4 (IQR 4.6–5.9) vs. 6.1 (IQR 5.4–6.9), n.s.) had no influence on the values during sepsis. Pretreatment with desipramine resulted in significantly higher GSH/GSSG ratios in SMPD1^−/−^ animals at 24 h following sepsis as compared to SMPD1^+/+^.

### 2.3. Lower Troponin I and Lactate Dehydrogenase (LDH) Levels Indicate Less Tissue Injury in Desipramine-Pretreated Animals

The abrogation of oxidative stress of animals pretreated with desipramine encouraged us to investigate a specific clinical marker of alterations in cardiomyocyte integrity, troponin I, and a global marker of tissue injury, lactate dehydrogenase (LDH).

**Figure 3:**
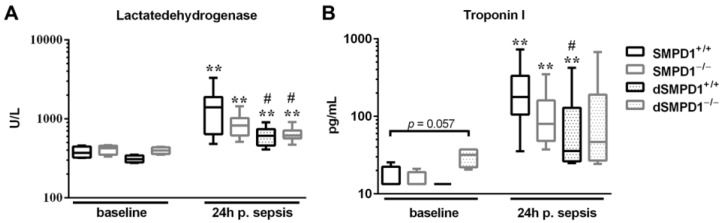
Desipramine pretreatment improves cardiac integrity in the acute phase of sepsis. Lactate dehydrogenase (LDH) as a global marker of tissue injury and troponin I levels as a cardiomyocyte specific marker in serum samples. (**A**) LDH was significantly increased in all strata, although less pronounced following desipramine pretreatment (dSMPD1^+/+^, dSMPD1^−/−^); (**B**) Troponin I levels increased in SMPD1^+/+^ as well as SMPD1^−/−^ animals (24 h), whereas desipramine-pretreated animals (dSMPD1^+/+^) demonstrated less pronounced enhancement as compared to SMPD1^+/+^. dSMPD1^−/−^ animals displayed no change in the values over time, but had significantly increased baseline levels. Data were obtained from *n* ≥ 4 at baseline and at least *n* ≥ 6 at 24 h. * *p* ≤ 0.05; ** *p* ≤ 0.01 versus corresponding baseline values; # *p* ≤ 0.05 versus SMPD1^+/+^ at the corresponding time points. Abbreviations: p. sepsis = post sepsis.

All strata had a significant increase of LDH levels following polymicrobial sepsis as compared to baseline (Figure 3A). Interestingly, desipramine pretreatment of SMPD1^−/−^ (612.0 (IQR 566.0–710.0) U/L, *p* ≤ 0.05) and SMPD1^+/+^ (610.0 (IQR 566.0–710.0) U/L, *p* ≤ 0.05) resulted in less pronounced elevation following sepsis induction as compared to untreated SMPD1^+/+^ animals (1395.0 (IQR 640.0–1885.0) U/L).

To further characterize heart specific impairment of cell integrity, we measured troponin I levels in serum samples. Sepsis resulted in a strong increase of troponin I serum levels in SMPD1^+/+^ in the acute phase (baseline: 13.3 (IQR 13.3–22.4) vs. 177.2 (IQR 105.2–332.6) pg/mL, *p* ≤ 0.01; Figure 3B). Furthermore, SMPD1^−/−^ animals displayed enhanced levels of troponin I as compared to corresponding baseline animals (baseline: 13.3 (IQR 13.3–19.02) vs. 79.6 (48.1–160.1) pg/mL, *p* ≤ 0.01). Desipramine pretreatment in dSMPD1^−/−^ animals led to increased levels at baseline (31.7 (IQR 22.0–37.2) pg/mL, *p* = 0.057), but remained unchanged during sepsis (46.5 (IQR 26.7–190.1) pg/mL). Strikingly, dSMPD1^+/+^ animals demonstrated significantly lower levels as compared to SMPD1^+/+^ in the acute phase [35.5 (IQR 26.2–128.8) pg/mL, *p* ≤ 0.05].

### 2.4. Dysregulated Cardiac Metabolism of Ceramide Is Independent of Acid Sphingomyelinase in the Acute Phase of Sepsis

**Figure 4:**
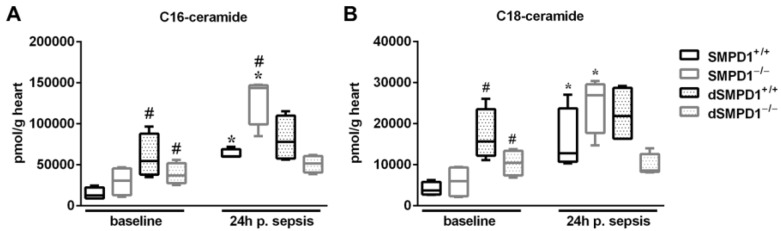
Dysregulated C16/C18-ceramide metabolism during sepsis. Ceramide specimens were analyzed in cardiac tissue homogenates. Data are obtained from *n* = 4 per strata/time point. (**A**) C16-ceramide and (**B**) C18-ceramide demonstrating increased generation in SMPD1^+/+^ and SMPD1^−/−^ animals, respectively, whereas dSMPD1^+/+^ as well as dSMPD1^−/−^ revealed enhanced levels at baseline, but unchanged values during sepsis. * *p* ≤ 0.05 versus corresponding baseline values; # *p* ≤ 0.05 versus SMPD1^+/+^ at corresponding time points. Abbreviations: p. sepsis = post sepsis.

C16-ceramide significantly increased in myocardial tissue homogenates in SMPD1^+/+^ from 12,629 (IQR 8900–22,254) pmol/g at baseline vs. 60,140 (IQR 59,697–68,968) pmol/g following polymicrobial sepsis (*p* ≤ 0.05, Figure 4A). Pretreatment of SMPD1^+/+^ animals with desipramine resulted in significantly elevated baseline values (54,425 (IQR 59,697–68,968) pmol/g, *p* ≤ 0.05 vs. SMPD1^+/+^ at baseline), but abrogated the increase of ceramide generation following sepsis (77,879 (IQR 57,644–109,723) pmol/g). Interestingly, induction of sepsis also increased C16-ceramide content in SMPD1^−/−^ animals (baseline: 30,467 (IQR 12,981–45,347) vs. 143,436 (IQR 99,296–146,714) pmol/g) as compared to corresponding baseline animals. However, pretreatment of SMPD1^−/−^ animals with desipramine resulted in complete abrogation of C16-ceramide increase (dSMPD1^−/−^: 36,999 (IQR 27,382–51,698) pmol/g), whereas similar as in SMPD1^+/+^, animals pretreated with desipramine showed already significantly increased baseline values (dSMPD1^+/+^: 51,466 (IQR 40,521–60,348) pmol/g).

Generation of C18-ceramide plays a key role in regulating cellular functions. In Figure 4B, a 3.85-fold increase of C18-ceramide in heart tissue homogenates of SMPD1^+/+^ was demonstrated in the acute phase of sepsis (baseline: 3726 (IQR 2731–5790) vs. 12,748 (IQR 10,695–23,677) pmol/g, *p* ≤ 0.05). SMPD1^−/−^ animals revealed a significantly enhanced generation of C18-ceramide (baseline: 5974 (IQR 2331–9287) vs. 26,929 (IQR 17,708–29,558) pmol/g, *p* ≤ 0.05), comparable to the results obtained with C16-ceramide. The pretreatment of SMPD1^+/+^ as well as SMPD1^−/−^ animals with desipramine significantly increased baseline values as compared to SMPD1^+/+^ (dSMPD1^+/+^: 15,633 (IQR 12,167–23,493) pmol/g heart; dSMPD1^−/−^: 10,465 (IQR 7356–13,338) pmol/g, *p* ≤ 0.05 each), respectively, but completely abrogated its generation due to sepsis (dSMPD1^+/+^: 21,832 (IQR 16,309–28,691) pmol/g; dSMPD1^−/−^: 8539 (IQR 8210–12,596) pmol/g; 24 h).

### 2.5. Gene Expression Analysis of Heart Tissue

**Figure 5:**
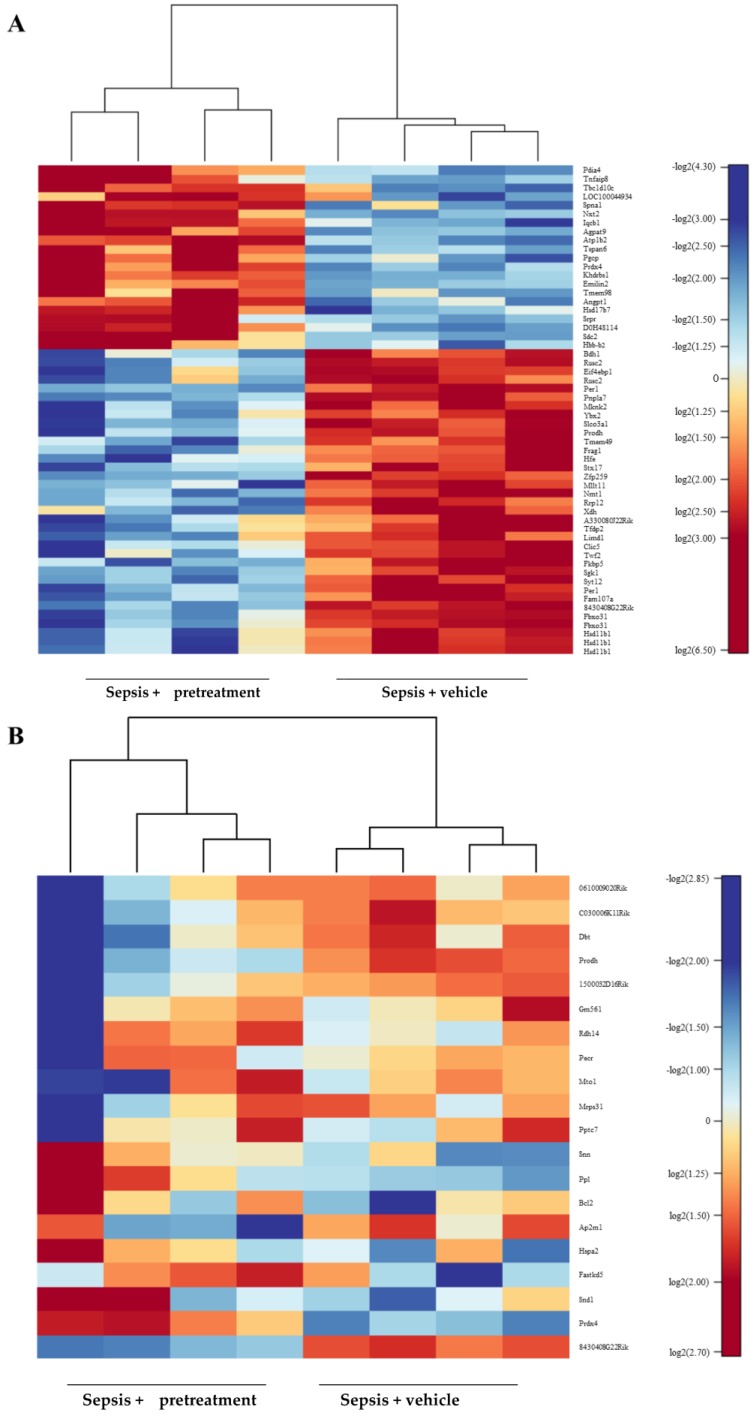
Analysis of differentially expressed transcripts from heart tissue homogenates comparing SMPD1^+/+^ and desipramine-pretreated SMPD1^+/+^ animals and sepsis. The transcriptional expression of 11,620 detected transcripts sampled at 24 h after polymicrobial sepsis were analyzed with the Illumina iScan microarray system. (**A**) Heatmap of differentially expressed transcripts in heart homogenates is demonstrated comparing SMPD1^+/+^ and dSMPD1^+/+^ (*n* = 4 each, columns). Relative expression changes due to sepsis are visualized by color code; (**B**) Subset of transcripts related to mitochondrial activity are visualized comparing SMPD1^+/+^ and dSMPD1^+/+^ following sepsis (*n* = 4 each, columns).

Due to the fact that desipramine pretreatment resulted in improved cardiac function in sepsis independent from acid sphingomyelinase, we found 11,620 gene transcripts of cardiac homogenates detectable comparing SMPD1^+/+^ and dSMPD1^+/+^, but 56 transcripts were found differentially expressed with False-Discovery-Rate (FDR) adjusted *p*-values ≤ 0.1 following polymicrobial sepsis, among others transcripts *TNFaip8*, *Atp1b2*, *Prdx4*, *Eif4ebp1*, and *Xdh* (Figure 5A). These transcripts included essential factors of cytokine response, apoptosis, antioxidantive response and heart muscle function (Table S1A). Furthermore, transcripts related to mitochondria revealed 20 transcripts, which were differentially expressed between SMPD1^+/+^ and dSMPD1^+/+^ (*p* ≤ 0.02, Figure 5B). Upregulation of transcripts, such as *Bcl2* and *Prdx4* in dSMPD1^+/+^ is known as a factor that contributes to an anti-oxidative response (Table S1B). Overall, desipramine pretreatment regulates gene expression of important factors contributing to attenuate septic cardiomyopathy.

**Figure 6:**
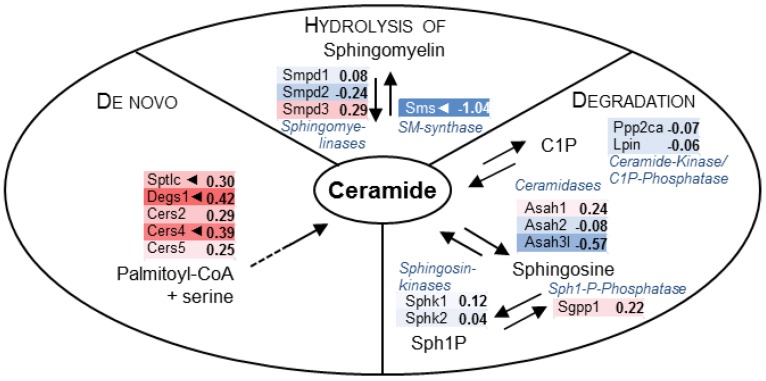
Ceramide metabolism in desipramine-pretreated mice undergoing septic cardiomyopathy. Data of transcripts related to biosynthesis and metabolism of ceramide were extracted from micro array experiments for schematic depiction of gene expression changes comparing mice with polymicrobial sepsis with subsequent cardiac dysfunction. Desipramine pretreatment not only resulted in inhibition of rapid and transient ceramide generation by acid sphingomyelinase *Smpd1*, but also in blockade of metabolism by ceramidases (*Asah1*, *Asah2*, *Asah3l*), which were all moderately down-regulated. The increase of ceramide levels could also be controlled by an up-regulation of transcripts related to de novo synthesis of ceramide: three ceramide synthases (*Cers2*, *Cers4*, *Cers5*) were found to be up-regulated. Interestingly, the desaturase *Degs1* catalyzing the last step in the ceramide biosynthetic pathway and a catalytic subunit of the rate limiting enzyme serine-palmitoyl transferase (*Sptlc*) were also significantly up-regulated. Development of ceramide overload might also be affected by strong and significant down-regulation of sphingomyelin synthase (*Sms*). Transcripts encoding enzymes for the metabolism of sphingosine (sphingosine kinases *Sphk1/2* as well as sphingosine-1-phosphate phosphatase, *Sgpp1*) were unaffected. There was also no change in transcript levels of enzymes regulating (de-)phosphorylation of ceramide (*Ppp2ca*, *Lpin*). Change of expression level is represented by a color code (blue down-regulation, red up-regulation), significant changes are indicated by an arrow (Abbreviations: C1P: ceramide-1-phosphate).

In depth analysis of transcripts known to play a part in regulation of tissue bound ceramide content resulted in clear evidence of up-regulation of genes regarding de novo synthesis (especially the rate limiting enzyme serine palmitoyltransferase, *Sptlc*), whereas genes encoding either Sphingomyelin-hydrolysis remained nearly unchanged or were found to be slightly down-regulated (Figure 6).

## 3. Discussion

In vivo sepsis-induced cardiac dysfunction was assessed by echocardiography and a significant reduction of cardiac output in a clinically relevant model of polymicrobial sepsis was documented. A moderate degree of impaired systolic contractility is in line with recent observations that at early time points an impaired systolic contractility is hard to document in 2- or 5-month-old mice, whereas 8-month-old mice exhibited severe functional impairment [34]. Despite the high incidence of sepsis in the elderly [35], we performed our study in young and healthy animals to eliminate a bias due to disease progression and manifestation of a Niemann–Pick phenotype in our genotype [36].

Here, we provide first evidence (1) of a dysregulated ceramide metabolism and (2) identified ceramide as a molecular key player in alterations of cardiomyocyte integrity and function during development of sepsis-induced cardiac dysfunction. Considering different origins of ceramides, our results demonstrate (3) increased intermediate-chain C16/C18-ceramide content in cardiac tissue homogenates due to increased de novo synthesis, but this was generated independently from the hydrolyzing activity of acid sphingomyelinase. Furthermore, we demonstrated (4) a potential therapeutic effect of desipramine, resulting in improved cardiac function as well as diminished oxidative stress and troponin I levels following polymicrobial sepsis.

An increased activity of SMPD1 has been observed in response to exogenous and endogenous mediators including oxidative stress, cytokines or invading microorganisms [37]. However, our experiments were based on the observation that increased plasma levels of SMPD1 have been found in patients with sepsis as well as patients with chronic heart failure [13,14,15,16,38]. Furthermore, numerous studies have described ceramide-induced caspase-3 and -8-like protease activation in cardiomyocytes [39] and cardiomyocyte apoptosis in ischemia/reperfusion injury. Amitriptyline, a functional inhibitor of SMPD1 as is desipramine, abrogated these effects and protected cardiomyocytes from damage in an ex vivo model [28]. Ceramides have been described as a mediator of Ca^2+^-load, controlling cardiomyocyte cell death [33]. Stress-induced activation of p38-kinase was abrogated by treatment with amitriptyline in a SMPD1-specific manner in neuronal cells [40]. The crucial role of ceramide is also supported by the observation of depressed maximal velocity of shortening as a consequence of acute in vitro exposure to ceramides which seems to be regulated by a specific isoform of protein kinase C (PKCε) [41].

### 3.1. Dysregulated Ceramide Metabolism in Sepsis-Induced Cardiac Dysfunction and Effects of Modulation

Our aim was to clarify the role of tissue specific ceramide generation during sepsis and additionally to find whether the enhanced generation is caused by SMPD1 as a common cardiac link also relevant in patients with heart failure. Following the induction of sepsis with an intraperitoneal administration of standardized fecal slurry, we observed a rapid and transient increase of both enzyme activity and C16/C18-ceramide content in cardiac tissue homogenates which was accompanied by corresponding changes in SMPD1-activity in SMPD1^+/+^ (data not shown). From a biophysical perspective, the distribution of sterols within sphingomyelin-containing membranes and the addition/generation of ceramides affects sterol partitioning in a chain-length dependent manner, since ceramides with intermediate chain lengths (i.e., C16/C18-ceramides) were the most effective in reducing sterol partitioning into the membrane influencing its lateral organization and forming separate ceramide-enriched domains [42,43]. Evaluating its role in stress response [44], we further detected significantly increased oxidative stress levels in cardiac tissue homogenates. Furthermore, LDH as a global marker for tissue injury and troponin I levels as a cardiac specific marker were increased in serum of SMPD1^+/+^ mice, reflecting both, injury of remote organs and of heart tissue.

Our data suggest a novel detrimental role of a rapid and transient ceramide generation in cardiomyocyte integrity during sepsis. Therefore, we tested whether a pharmacological modulation with desipramine can abrogate these effects and thus might be of therapeutic interest. Strikingly, pretreatment abrogated the increase of C16/C18-ceramide during sepsis, whereas ceramide content was already significantly increased at baseline. Desipramine is an FDA-approved antidepressant drug which has been in clinical use for decades to treat psychiatric and neurological disorders. Furthermore, it belongs to a family of functional inhibitors of SMPD1 (FIASMA), whereas it is also known to initiate the breakdown of several other intralysosomal enzymes involved in ceramide metabolism including ceramidase [30]. However, we found oxidative stress and troponin I levels to be significantly reduced following treatment with this compound. This was more so for troponin I which is associated with sepsis-induced cardiac dysfunction and mortality of patients with sepsis [5,45]. We identified numerous transcripts essentially involved in cytokine response (*Tnfaip8*), apoptosis (*Bcl2*), antioxidative response (*Prdx4*) as well as cardiac muscle function (*Xdh*) in whole heart homogenates in comparison to untreated SMPD1^+/+^. These results show a beneficial effect of desipramine at a transcriptional level, but the precise molecular mechanism needs further examination. Our data is in line with a report demonstrating a beneficial effect of amitriptyline on primary isolated human cardiomyocytes’ apoptosis in an ex vivo cardioplegia and reperfusion model [28]. In addition, ceramide treatment initiates the activation of a volume-sensitive chloride current which influences cardiac electro-mechanical activity and cell volume as well as apoptosis [25]. A panel of pathways has been discussed as potentially significant in triggering mitochondrial dysfunction in SCD, although it remains controversial whether mechanisms impair mitochondrial function or serve to restore mitochondrial function [46]. In line with those data, we also identified a differential expression pattern of transcripts associated with mitochondrial function after pretreatment.

### 3.2. Pleiotropic Role of Ceramide Formation and Mechanisms of Stress Response

We induced sepsis in SMPD1^−/−^ animals to identify enzyme contribution to tissue-specific ceramide generation [47]. Numerous SMPD1-deficient cell types including hepatocytes are resistant to TNF-α and FAS-ligand induced apoptosis which could be both overcome by treating with ceramide [48,49]. Unexpectedly, our data revealed increasing ceramide levels in homogenates of SMPD1^−/−^ animals, suggesting a dysregulated ceramide metabolism independent of SMPD1. Besides acid sphingomyelinase, there are other isoforms (e.g., neutral sphingomyelinase) as well as two other pathways contributing to ceramide metabolism in health and disease: de novo synthesis and the salvage pathway [19]. Accordingly, confirming enhanced ceramide generation, SMPD1^−/−^ animals also displayed increased oxidative stress values and increased levels of troponin I in the acute phase of sepsis.

Beyond rapid formation of ceramide triggered by sphingomyelin hydrolysis, there is evidence of increased de novo synthesis after pro-inflammatory stimulation and during post-ischemic myocardial injury [50]. Inhibition of the rate-limiting step of ceramide biosynthesis (Serine Palmitoyltransferase 1 (Sptlc1), Figure 6) exerts protective effects, whereas use of functional inhibitors of SMPD1 such as desipramine also improved cardiac function and prevented postischemic injury [28]. The exact role of varying concentrations and kinetic/dynamic of tissue bound ceramide has to be further elucidated in future studies.

To further clarify the role of pharmacological action of desipramine beyond sphingomyelinase inhibition, we also pretreated SMPD1^−/−^ animals. Interestingly, in these animals desipramine pretreatment significantly increased baseline ceramide content similar to that in dSMPD1^+/+^ by unknown mechanisms and also abrogated the increase of ceramide, oxidative stress, LDH and troponin I. With respect to our findings in SMPD1^−/−^ and dSMPD1^−/−^ animals, we decreased acid sphingomyelinase as the only enzyme responsible for ceramide control in response to sepsis in cardiac tissue. In consideration of the detrimental role of ceramides on cardiomyocyte integrity and apoptosis [28], the abrogation of an increase of ceramides independent of the baseline values seems to be important for improvement of cardiac integrity and function. Interestingly, there is increasing evidence that ischemic preconditioning protects from apoptosis and reduces the infarct size in ischemia/reperfusion [51]. Preconditioning partially involved alterations in sphingolipid composition, in particular ceramide [51]. Interestingly, exogenous ceramide treatment was capable of mimicking the effects of preconditioning with subsequent reduction of infarct size [52,53,54]. As we pretreated animals with desipramine for seven days it might be speculated that ceramide content and de novo synthesis was increased at baseline due to inhibition of degrading enzymes, thus resulting in preconditioning effects. Both of these phenomena triggered by desipramine, i.e., inhibition of overwhelming ceramide peak concentrations and preconditioning, might contribute to improved cardiac outcome in both strata following polymicrobial sepsis. Therefore, additional experiments are needed to investigate the role of preconditioning in cardiomyocytes in sepsis.

### 3.3. Critical Review of Chosen Model and Mode of Pharmacological Intervention

A reduction of cardiac sympathetic transmitters as observed in congestive heart failure, can be induced by exogenous administration of norepinephrine (NE) [55,56]. However, depletion of NE might be inhibited by desipramine, which is known to function as a reuptake inhibitor of NE in ex-vivo ventricular preparations [56,57]. The dosage of desipramine we applied is well tolerated and caused no adverse clinical or hemodynamic effects. In an in vivo study of chronic heart failure using a similar dosage, desipramine was shown to increase myocardial NE in both sham-operated and chronic heart failure animals. The drug caused NE uptake inhibition as evidenced by an exaggerated pressor response and interstitial NE increase following its infusion, but no effects on cardiac function or hemodynamics in rabbits with left ventricular dilation and dysfunction [58]. This supports the concept of a ceramide-independent action of desipramine for cardio-protection. In an in vitro model of NE-treated phaeochromocytoma cells, desipramine abolished reduction of tyrosine hydroxylase, the rate-limiting enzyme of NE synthesis and NE-induced cell death [58]. In addition, desipramine has been shown to exert sympatho-inhibitory effects via stimulation of presynaptic α-2-receptors [59], but also desensitization was observed [60]. Even though there is no exact definition of septic cardiomyopathy or sepsis-induced cardiac dysfunction, it is well documented that systolic and diastolic myocardial function are of utmost importance in sepsis patients. Decreased ejection fraction and cardiac output appear to be reversible concomitants following the acute phase. These phenomena are a result of a complex mixture of pathophysiological alterations including hemodynamic factors and molecular and structural alterations [61]. Furthermore, it has been demonstrated that cardiac dysfunction results in worse outcomes for these patients [2]. Transthoracic echocardiographic (TTE) analysis is a non-invasive, commonly used method with high sensitivity to quantify myocardial function in septic patients [62]. Therefore, we used TTE to investigate cardiac dysfunction in our strata. TTE analysis revealed improved ventricular function represented by ejection fraction as well as cardiac output and less pronounced diastolic dysfunction (E’, MV E/A ratio) as compared with untreated SMPD1^+/+^ as well as SMPD1^−/−^.

### 3.4. Conclusions

We conclude that increased ceramide generation plays an essential and decisive role in cardiac integrity and dysfunction during the acute phase of sepsis. Furthermore, the dysregulated ceramide metabolism in cardiac tissue increases oxidative stress as well as troponin I levels. Strikingly, pretreatment with the FDA-approved drug desipramine abrogates ceramide increase, oxidative stress, troponin I elevation, and finally improves cardiac dysfunction. Desipramine pretreatment is able to adjust pathophysiological cardiac alterations during sepsis, nevertheless an effect of treatment following triggering of host response has to be substantiated in further studies. On a molecular level, de novo synthesis of ceramide upon desipramine treatment seems to function as a critical factor for the observed adaptive cellular response.

While cardiomyopathy during sepsis is responsible for considerable morbidity and mortality, there is an urgent need for development of specific stratagems extending causal and supportive therapy of the underlying disease. Here, our translational animal study identifies the role of modulation of the lipid mediator ceramide as a novel and protective step to influence reduced myocardial function and cellular integrity. Besides implicating therapeutic potential for targeting ceramide formation, our data additionally emphasize that administration of FDA-approved drugs, known to affect this process, might be a promising therapeutic strategy to counteract the sequelae of septic cardiomyopathy. Discontinuation of these compounds during intensive care unit therapy may result in adverse effects. Further evaluation is needed to investigate the mechanisms involved in the increase of ceramide generation in the heart as well as its role in preconditioning effects. To clarify the role of desipramine as a therapeutic option in septic cardiomyopathy, further preclinical and observational studies analyzing septic patients undergoing treatment with FIASMA for treatment of neurological disorders are needed.

## 4. Methods and Material

### 4.1. Animals

Transgenic mice (C57/BL6 background) with a complete deficiency in SMPD1 function (SMPD1^−/−^) and their wild type (SMPD1^+/+^) littermates (aged 8–12 weeks) were used in this study [63]. For each experiment, similar proportions of male and female mice were randomly selected, maintained under artificial day–night conditions at room temperature, and received a standard diet and water ad libitum. All experiments were performed in accordance with the German legislation on protection of animals and with permission of the regional animal welfare committee (Thueringer Landesamt fuer Lebensmittelsicherheit und Verbraucherschutz, 02-009/12, permission: 2012); the investigation conforms with the Guide For the Care and Use of Laboratory Animals, published by the US National Institutes of Health in its latest version (8th edition, update 2011).

### 4.2. Desipramine Pretreatment and Sepsis Model

Randomly selected SMPD1^+/+^ and SMPD1^−/−^ animals were treated seven days with desipramine hydrochloride (isotonic saline solution, 20 mg/kg body weight) every 24 h subcutaneously prior to induction of polymicrobial infection, which was continued up to euthanasia. Sepsis was induced by the standardized peritoneal contamination and infection model [32,47]. Briefly, intraperitoneal injection of fecal slurry (diluted 1:4 in saline solution) was performed (3.0 µL/g B.W.) into the right lower quadrant of the abdomen with a 21-gauge cannula. Without supportive treatment, severity of the insult might result in a 100% mortality rate within 24 h (data not shown). However, administration of antibiotics (20 mg/kg meropenem) starting 6 h every 24 h during host response rescued animals and adjusted survival rate to 60%–70% during observation period of four days. All animals were treated with physiological saline solution in a total volume of 25 µL/g B.W. subcutaneously twice daily in due consideration of desipramine and meropenem administration. Disease progression and outcome of mice during host response was continuously assessed using the clinical severity scores (CSS) (data not shown) as previously described [32]. Ultimately, animals were deeply anesthetized prior to necropsy (isoflurane 2%).

### 4.3. Mass Spectrometry

Fresh frozen heart tissue homogenates from each stratum (SMPD1^+/+^, SMPD1^−/−^, dSMPD1^−/−^, dSMPD1^+/+^; *n* = 4/stratum) at baseline and 24 h following septic insult were analyzed with respect to C16- and C18-ceramide content. Measurements were performed according to protocol using liquid chromatography coupled to triple-quadrupole mass spectrometry as previously described [64]. For detection, the QTrap triple-quadrupole mass spectrometer (ABSciex) interfaced with the Merck-Hitachi Elite LaChrom series 3.1.3 chromatograph and autosampler (VWR) was used and analyzed using Analyst 1.4 (ABSciex). 

### 4.4. GSH and GSSG

Glutathione, in its reduced (GSH) and oxidized (GSSG) state, was determined according to Ellman [65] and Hissin and Hilf [66]. Briefly, fresh frozen samples were processed and measured as previously reported [67]. For analysis, data were obtained from *n* = 4 from each stratum and time point (SMPD1^+/+^, SMPD1^−/−^, dSMPD1^+/+^, dSMPD1^−/−^).

### 4.5. Laboratory Marker

Troponin I was measured in 1:10 diluted serum samples by a commercially available ELISA kit (USCN, Life science Inc., Wuhan, China). The procedure was followed to manufacturer’s instructions. Lactate dehydrogenase was measured using the clinical chemistry analyzer Fuji Dri-Chem 3500i (Sysmex, Leipzig, Germany) according to manufacturer’s instructions. For analysis, data were obtained from *n* = 4 at baseline and at least *n* ≥ 7 animals following polymicrobial sepsis from each stratum and time point (SMPD1^+/+^, SMPD1^−/−^, dSMPD1^+/+^, dSMPD1^−/−^).

### 4.6. Gene Expression Analysis

Cardiac tissues were collected from untreated SMPD1^+/+^ animals and desipramine-pretreated SMPD1^+/+^ 24 h following sepsis induction (*n* = 4/stratum). Samples were analyzed by pangenomic microarray mouseWG-6 v1.1 expression bead chips using an iScan platform (Illumina, San Diego, CA, USA) measuring the variation of expression rate of >42,000 transcripts. Biostatistical analysis and validation of expression were performed.

### 4.7. Transthoracic Echocardiography

Animals were anesthetized with isoflurane (2%) and examined in supine position using a Vevo 770 High-Resolution Imaging System (Visual Sonics, Toronto, Canada). Two-dimensional short-axis views of the left ventricle at papillary muscle level were obtained. Two-dimensional guided M-mode tracings were recorded. The main echocardiographic parameters were measured, including heart rate (HR), left ventricular end diastolic dimension (LVEDD) and left ventricular wall thickness in diastole and systole (LV wall). Based on these measurements, ejection fraction (EF) and cardiac output were calculated. The pulsed Doppler of the left ventricular inflow (E and A waves) was assessed in the apical four-chamber view, and the sample volume was placed at the mitral tip level. Pulse-wave tissue Doppler was obtained from the four-chamber view, and the sample volume was placed on the basal interventricular septum. The early and late diastolic waves (E′ and A′) were detected and their peak velocities were measured [68].

### 4.8. Statistics

For statistical analysis the Mann-Whitney U-test was performed to evaluate significant differences between strata and time points, since data were not normally distributed. Levels of *p* ≤ 0.05 were considered to be of statistical significance. 

## 5. Conclusions

We conclude that increased ceramide generation plays an essential and decisive role in cardiac integrity and dysfunction during the acute phase of sepsis. Furthermore, the dysregulated ceramide metabolism in cardiac tissue increases oxidative stress as well as troponin I levels. Strikingly, pretreatment with the FDA approved drug desipramine abrogates ceramide increase, oxidative stress, troponin I elevation, and finally improves cardiac dysfunction. Desipramine pretreatment is able to adjust pathophysiological cardiac alterations during sepsis, nevertheless an effect of treatment following triggering of host response has to be substantiated in further studies. On a molecular level, de-novo synthesis of ceramide upon desipramine treatment seems to function as a critical factor for the observed adaptive cellular response.

While cardiomyopathy during sepsis is responsible for considerable morbidity and mortality, there is an urgent need for development of specific stratagems extending causal and supportive therapy of the underlying disease. Here, our translational animal study identifies the role of modulation of the lipid mediator ceramide as a novel and protective step to influence reduced myocardial function and cellular integrity. Besides implicating therapeutic potential for targeting ceramide formation, our data additionally emphasize that administration of FDA-approved drugs, known to affect this process, might be a promising therapeutic strategy to counteract the sequelae of septic cardiomyopathy. Discontinuation of these compounds during intensive care unit therapy may result in adverse effects. Further evaluation is needed to investigate the mechanisms involved in the increase of ceramide generation in the heart as well as its role in preconditioning effects. To clarify the role of desipramine as a therapeutical option in septic cardiomyopathy, further preclinical and observational studies analyzing septic patients undergoing treatment with FIASMA for treatment of neurological disorders are needed.

## Supplementary Materials

Supplementary materials can be found at www.mdpi.com/1422-0067/18/4/839/s1.
