# Supplementary material for: Adjustment of Dysregulated Ceramide Metabolism in a Murine Model of Sepsis-Induced Cardiac Dysfunction
Source: Int J Mol Sci. 2017 Apr 15;18(4):839. doi: 10.3390/ijms18040839 (PMC5412423; doi:10.3390/ijms18040839)
Supplement: Supplementary file 1 [file ijms-18-00839-s001.pdf]

# Supplementary material

## Adjustment of Dysregulated Ceramide Metabolism in a Murine Model of Sepsis-Induced Cardiac Dysfunction

**Ha-Yeun Chung [1,2], Anna S. Kollmey [1,2], Andrea Schrepper [1,3], Matthias Kohl [4], Markus F. Blaess [2], Sebastian N. Stehr [1,5], Amelie Lupp [6], Markus H. Gräler [1,2] and Ralf A. Claus [1,2,*]**

[1] Center for Sepsis Control and Care, Jena University Hospital, Am Klinikum 1, 07747 Jena, Germany; Ha-yeun.Chung@med.uni-jena.de (H.-Y.C.); sophia.kollmey@web.de (A.S.K.); Andrea.Schrepper@med.uni-jena.de (A.S.); sebastian.stehr@medizin.uni-leipzig.de (S.N.S.); markus.graeler@med.uni-jena.de (M.H.G.)
[2] Department of Anesthesiology and Intensive Care, Jena University Hospital, Am Klinikum 1, 07747 Jena, Germany; markus.blaess@med.uni-jena.de
[3] Department of Cardiothoracic Surgery, Jena University Hospital, Am Klinikum 1, 07747 Jena, Germany.
[4] Institute of Precision Medicine, Furtwangen University, 78054 Villingen-Schwenningen, Germany; matthias.kohl@stamats.de
[5] Department of Anesthesiology and Intensive Care, University of Leipzig, Liebigstrasse 20, 04103 Leipzig, Germany
[6] Institute of Pharmacology and Toxicology, Jena University Hospital, Drackendorfer Straße 1, 07747 Jena, Germany; amelie.lupp@med.uni-jena.de

### Legends to Tables and Figure

**Table 1: Functional description of differentially expressed genes (DEG) (A)** Alternative symbols and (myocardial) function representing DEG. **(B)** Alternative symbols and (mitochondrial) function representing DEG.

# Table 1A

| ***Symbol*** | **Alternative symbols and (myocardial) function representing DEG (Fig 5A)** |
|---|---|
| *Pdia4* | PROTEIN O-MANNOSYLTRANSFERASE 2; POMT2: encodes an integral membrane protein of the endoplasmic reticulum (ER) sharing significant sequence similarity with a family of protein O-mannosyltransferases of *S. cerevisiae*. POMT1 and POMT2 proteins catalyze the synthesis of the O-mannosyl glycan found on alpha-dystroglycan |
| *Tnfaip8* | TUMOR NECROSIS FACTOR-ALPHA-INDUCED PROTEIN 8; TNFAIP8: early response gene, with expression peaking between 1.5 and 3 hours |
| *Tbc1d10c* | TBC1 DOMAIN FAMILY, MEMBER 10C; TBC1D10C: syn Carabin, endogenous inhibitor of calcineurin that also inhibits the Ras signaling pathway through its intrinsic Ras GTPase-activating protein activity |
| *Nxt2* | NTF2-LIKE EXPORT FACTOR 2: nuclear pore complex-associated protein for export of mRNAs from the nucleus; maintaining morphogenetic integrity of embryonic heart (1) |
| *Iqcb1* | IQ MOTIF-CONTAINING PROTEIN B1; IQCB1: interaction with calmodulin |
| *Agpat9* | 1-ACYLGLYCEROL-3-PHOSPHATE O-ACYLTRANSFERASE 9; AGPAT9: catalyzes the initial step of *de-novo* triacylglycerol (TAG) synthesis by converting glycerol-3-phosphate (G3P) to lysophosphatidic acid (LPA); strongly induced expression by treatment with DNA-hypomethylating agent 5-azacytidine, promoting triacylglycerol synthesis and cytosolic lipid droplet formation (2) |
| *Atp1b2* | ATPase, Na+/K+ TRANSPORTING, BETA-2 POLYPEPTIDE; ATP1B2: plasma membrane pump with numerous physiologic functions. It maintains ionic homeostasis that is critical for cell survival, differentiation, and apoptosis. Beta subunits, such as ATP1B2, are responsible for formation and structural integrity of the Na+/K+ ATPase holoenzyme |
| *Tspan6* | TETRASPANIN 6; TSPAN6: Tetraspanins are frequently expressed at the cell surface in association with each other and with other molecules, such as integrins; regulating cell adhesion, migration, proliferation, and differentiation; negative regulator of the retinoic acid-inducible gene I-like receptors pathway in a ubiquitination-dependent manner (3) |
| *Prdx4* | PEROXIREDOXIN 4: Thioredoxin peroxidase regulating NF-κB activation; interaction with HIF-1a following hypoxia impairing HIF-binding to the hypoxia response elements of a subset of HIF target genes (4) |
| *Khdrbs1* | KH DOMAIN-CONTAINING, RNA-BINDING, SIGNAL TRANSDUCTION-ASSOCIATED PROTEIN 1: evolutionarily conserved signal transduction activator of RNA (STAR) family of RNA-binding proteins. These proteins play key roles during cell differentiation and development |
| *Emilin2* | ELASTIN MICROFIBRIL INTERFACER 2; EMILIN2: expression during cardiovascular development, on cardiac stem cells, and in heart tissue in animal models of heart disease; influencing platelet aggregation induced by adenosine diphosphate, collagen, and thrombin (5) |
| *Angpt1* | ANGIOPOIETIN 1; ANGPT1: an angiogenic growth factor with antipermeability and antiinflammatory properties |
| *Hsd17b7* | 17-BETA-HYDROXYSTEROID DEHYDROGENASE VII; HSD17B7: biosynthesis of sex steroids and as a 3-ketosteroid reductase > EC 1.1.1.270< in the biosynthesis of cholesterol |
| *Sdc2* | SYNDECAN 2; SDC2: involved in development of a normal vasculature, tissue repair and angiogenesis; shed from the cell surface resulting in the release of its extracellular core protein inhibiting endothelial cell migration (6) |
| *Hbb-b2* | HEMOGLOBIN--BETA LOCUS; HBB: the alpha (HBA1, HBA2) and beta (HBB) loci determine the structure of the 2 types of polypeptide chains in adult hemoglobin, HbA |
| *Bdh1* | 3-HYDROXYBUTYRATE DEHYDROGENASE 1; BDH1: key enzyme in the ketone oxidation pathway, increased heart failure; marker of biochemical shift to ketone bodies as a significant fuel source for oxidative ATP production in the hypertrophied and failing heart (7) |
| *Rusc2* | RUN AND SH3 DOMAIN-CONTAINING 2; RUSC2: regulating stabilization of GIT2, a G-protein-coupled receptor kinase complex involved in diverse cellular processes such as cytoskeletal dynamics, membrane trafficking and focal adhesion turnover; control of Golgi reorientation and directional migration (8) |

| | |
|---|---|
| *Eif4ebp1* | EUKARYOTIC TRANSLATION INITIATION FACTOR 4E-BINDING PROTEIN 1; EIF4EBP: substrate of mTOR kinase activity; regulating sepsis-induced decrease in skeletal muscle protein synthesis (9) |
| *Pnpla7* | PATATIN-LIKE PHOSPHOLIPASE DOMAIN-CONTAINING PROTEIN 7; phospholipase involved in regulation of adipocyte differentiation; induction by metabolic stimuli |
| *Mknk2* | MITOGEN-ACTIVATED PROTEIN KINASE-INTERACTING SERINE/THREONINE KINASE 2; MKNK2: upregulated by palmitic acid affecting the expression of acetyl-CoA carboxylases-1 and fatty acid synthase in hepatocytes |
| *Slco3a1* | SOLUTE CARRIER ORGANIC ANION TRANSPORTER FAMILY, MEMBER 3A1; SLCO3A1: OATP3A1-V1 and -V2 transported prostaglandins E1 and E2, thyroxine, a cyclic endothelin receptor peptide antagonist, and vasopressin |
| *Prodh* | PROLINE DEHYDROGENASE (OXIDASE) 1; PRODH: is involved in the degradation of the amino acid proline. It catalyzes the conversion of proline to pyrroline-5-carboxylate |
| *Frag1* | ATPASE FAMILY AAA DOMAIN CONTAINING, MEMBER 5; ATAD5: DNA damage response protein involved in DNA repair following exposure to various genotoxic stresses |
| *Hfe* | HFE GENE; HFE: forms stable complexes with the transferrin receptor; complexes with TFRC, increasing the dissociation constant of transferrin (TF) for its receptor 10-fold. HFE does not remain at the cell surface, but traffics with TFRC to transferrin-positive internal compartments; appears to reduce cellular acquisition of iron from TF within endocytic compartments |
| *Stx17* | SYNTAXIN 17; STX17: STX17 and ATG14 are extensively colocalized at ER–mitochondria contact sites under starvation conditions; STX17 binds ATG14 and recruits to the ER–mitochondria contact site. The autophagosome-formation marker ATG5 also localizes at the site until formation is complete. STX is found to be involved into organelle biogenesis by demonstrating that the ER–mitochondria contact site is important in autophagosome formation (10) |
| *Nmt1* | N-MYRISTOYLTRANSFERASE 1; NMT1: catalyzing the transfer of myristate from CoA to proteins. *N*-myristoylation appears to be irreversible and is required for full expression of the biologic activities of *N*-myristoylated proteins; |
| *Rrp12* | RIBOSOMAL RNA PROCESSING 12 HOMOLOG; RPR12: Biogenesis, maturation and transport of small ribosomal subunits |
| *Xdh* | XANTHINE DEHYDROGENASE; XDH: oxidative metabolism of purines; important source of oxygen free radicals and related postischemic injury following conversion to xanthine oxidase (XO) by oxidation of sulfhydryl residues or by proteolysis. XDH-mediated oxygen radical generation markedly depressed $Ca^{2+}$-ATPase activity of isolated sarcoplasmic reticulum vesicles from cardiac muscle |
| *Limd1* | LIM DOMAIN-CONTAINING PROTEIN 1; LIMD1: The LIM motif had been previously identified in many developmentally important factors from various eukaryotes. These factors had been shown to play a role in intracellular signaling, transcriptional regulation, and cellular differentiation during development; The interaction between LIMD1 and RB1 inhibited E2F-mediated transcription and suppressed expression of a majority of genes with E2F1-responsive elements |
| *Clic5* | CHLORIDE INTRACELLULAR CHANNEL 5; CLIC5: plays a direct role in regulating mitochondrial reactive oxygen species generation; localized to cardiac mitochondria modulating mitochondrial function (11) |
| *Twf2* | TWINFILIN, DROSOPHILA, HOMOLOG OF, 2; TWF2: filament end barbing protein: binding actin monomers and capping protein, as well as efficiently capping actin filament barbed-ends (12) |
| *Fkbp5* | FK506-BINDING PROTEIN 5; FKBP5: Immunophilin with high affinity to immunosuppressive drugs rapamycin and FK520 |
| *Syt12* | SYNAPTOTAGMIN 12; SYT12: regulation of transmitter release |
| *Hsd11b1* | 11-BETA-HYDROXYSTEROID DEHYDROGENASE, TYPE I; HSD11B1: There are at least 2 isoforms of 11-beta-HSD. HSD11B1 was purified and cloned from rat liver. The glycoprotein enzyme (also called type I) catalyzes both 11-beta-dehydrogenation and the reverse 11-oxoreduction reaction. The enzyme and corresponding mRNA have been detected in a wide range of rat and human tissues, including liver, lung, and testis. |

**Table 1B:**

| *Symbol* | **Alternative symbols and (mitochondrial) function representing DEG (Fig. 5B)** |
|---|---|
| *Dbt* | DIHYDROLIPOAMIDE BRANCHED CHAIN TRANSACYLASE: catabolism of branched-chain amino acids (isoleucine, leucine, and valine) |
| *Prodh* | PROLINE OXIDASE: degradation of the amino acid proline; catalyzes the conversion of proline to pyrroline-5-carboxylate |
| *Rdh14* | RETINOL DEHYDROGENASE 14: microsomal enzyme; member of short-chain dehydrogenase/ reductase gene superfamily recognizing retinoids but not steroids; deficient beta-carotene metabolism is associated with cardiac dysfunction and increase of myocardial ceramides (13) |
| *Pecr* | PEROXISOMAL TRANS-2-ENOYL-COA REDUCTASE: chain elongation of fatty acids |
| *Mto1* | MITOCHONDRIAL TRANSLATION OPTIMIZATION 1, S. CEREVISIAE, HOMOLOGUE OF: highly conserved enzyme involved in tRNA modification and protein synthesis; MTO1-deficient mouse mirrors the human phenotype showing complex I defect and cardiomyopathy (14) |
| *Mrps31* | MITOCHONDRIAL RIBOSOMAL PROTEIN 31: member of the own mitochondrial translation machinery for production of 13 inner membrane proteins essential for oxidative phosphorylation (small subunit of the mitochondrial ribosome) |
| *Pptc7* | PHOSPHATASE PTC7: regulator of mitochondrial metabolism; involved in co-enzyme Q biosynthesis; enhancing oxidative stress resistance (15) |
| *Snn* | STANNIN: highly conserved, early responsive, cytoprotective gene; sensor of mitochondrial damage (16) |
| *Pp1* | PROTEIN PHOSPHATASE 1, CATALYTIC SUBUNIT, ALPHA ISOFORM: one of 4 major cytosolic protein phosphatases identified of eukaryotic cells that are responsible for dephosphorylation of serine and threonine residues in proteins; increased activity and subsequent enhanced protein dephosphorylation may play a role in diabetes-induced cardiac dysfunction (17) |
| *Bcl2* | B-CELL LYMPHOMA 2: integral inner mitochondrial membrane, interfering with programmed cell death independent of promoting cell division; i.v. administration of rec. protein in sepsis is improving survival and reducing number of apoptotic cells in the heart (18) |
| *Ap2m1* | ADAPTOR-RELATED PROTEIN COMPLEX 2, MU-1 SUBUNIT: component of the AP2 coat assembly protein complex of clathrin-coated vesicles; required for assembly/activity of vacuolar ATPase held responsible for proton pumping in acidification of endosomes and lysosomes. |
| *Hspa2* | HEAT SHOCK 70-KD PROTEIN 2: Nucleotide binding; ATP binding; unfolded protein binding/response to unfolded protein |
| *Fastkd5* | FAST KINASE DOMAIN CONTAINING PROTEIN 5: mitochondrial ribosome biogenesis, posttranscriptional RNA processing and biogenesis of mitochondrial ribosomes (19) |
| *Prdx4* | PEROXIREDOXIN 4: Negative regulation of oxygen and reactive oxygen species metabolic process; thioredoxin peroxidase regulating NF-κB activation (20) |

## 6. References


1. Huang H, Zhang B, Hartenstein PA, Chen JN, Lin S. (2005) NXT2 is required for embryonic heart development in zebrafish. *BMC developmental biology* **5:** 7.
2. Poirier S*, et al.* (2014) The epigenetic drug 5-azacytidine interferes with cholesterol and lipid metabolism. *J Biol Chem* **289:** 18736-18751.
3. Wang Y*, et al.* (2012) Tetraspanin 6 (TSPAN6) negatively regulates retinoic acid-inducible gene I-like receptor-mediated immune signaling in a ubiquitination-dependent manner. *J Biol Chem* **287:** 34626-34634.
4. Luo W*, et al.* (2016) PRDX2 and PRDX4 are negative regulators of hypoxia-inducible factors under conditions of prolonged hypoxia. *Oncotarget* **7:** 6379-6397.
5. Huang M*, et al.* (2015) EMILIN2 regulates platelet activation, thrombus formation, and clot retraction. *PloS one* **10:** e0115284.
6. De Rossi G*, et al.* (2014) Shed syndecan-2 inhibits angiogenesis. *J Cell Sci* **127:** 4788-4799.
7. Aubert G*, et al.* (2016) The Failing Heart Relies on Ketone Bodies as a Fuel. *Circulation* **133:** 698-705.
8. Duan B*, et al.* (2016) EGF-stimulated activation of Rab35 regulates RUSC2-GIT2 complex formation to stabilize GIT2 during directional lung cancer cell migration. *Cancer letters* **379:** 70-83.
9. Steiner JL*, et al.* (2014) Disruption of genes encoding eIF4E binding proteins-1 and -2 does not alter basal or sepsis-induced changes in skeletal muscle protein synthesis in male or female mice. *PloS one* **9:** e99582.
10. Hamasaki M*, et al.* (2013) Autophagosomes form at ER-mitochondria contact sites. *Nature* **495:** 389-393.
11. Ponnalagu D*, et al.* (2016) Molecular identity of cardiac mitochondrial chloride intracellular channel proteins. *Mitochondrion* **27:** 6-14.
12. Nevalainen EM, Skwarek-Maruszewska A, Braun A, Moser M, Lappalainen P. (2009) Two biochemically distinct and tissue-specific twinfilin isoforms are generated from the mouse Twf2 gene by alternative promoter usage. *Biochem J* **417:** 593-600.
13. Lee SA*, et al.* (2014) Cardiac dysfunction in beta-carotene-15,15'-dioxygenase-deficient mice is associated with altered retinoid and lipid metabolism. *Am J Physiol Heart Circ Physiol* **307:** H1675-1684.
14. Becker L*, et al.* (2014) MTO1-deficient mouse model mirrors the human phenotype showing complex I defect and cardiomyopathy. *PloS one* **9:** e114918.
15. Martin-Montalvo A*, et al.* (2013) The phosphatase Ptc7 induces coenzyme Q biosynthesis by activating the hydroxylase Coq7 in yeast. *J Biol Chem* **288:** 28126-28137.
16. Billingsley ML*, et al.* (2006) Functional and structural properties of stannin: roles in cellular growth, selective toxicity, and mitochondrial responses to injury. *Journal of cellular biochemistry* **98:** 243-250.
17. Rastogi S, Sentex E, Elimban V, Dhalla NS, Netticadan T. (2003) Elevated levels of protein phosphatase 1 and phosphatase 2A may contribute to cardiac dysfunction in diabetes. *Biochim Biophys Acta* **1638:** 273-277.
18. Iwata A*, et al.* (2011) Extracellular administration of BCL2 protein reduces apoptosis and improves survival in a murine model of sepsis. *PloS one* **6:** e14729.
19. Antonicka H, Shoubridge EA. (2015) Mitochondrial RNA Granules Are Centers for Posttranscriptional RNA Processing and Ribosome Biogenesis. *Cell reports*.
20. Li W*, et al.* (2012) Proteomic analysis of metabolic, cytoskeletal and stress response proteins in human heart failure. *Journal of cellular and molecular medicine* **16:** 59-71.
